# Gestational heat stress alters skeletal muscle gene expression profiles and vascularity in fetal pigs in a sexually dimorphic manner

**DOI:** 10.1186/s40104-022-00730-2

**Published:** 2022-07-15

**Authors:** Weicheng Zhao, Mark P. Green, Christina D. Marth, Fan Liu, Hieu H. Le, Gordon S. Lynch, Alan W. Bell, Brian J. Leury, Frank R. Dunshea, Jeremy J. Cottrell

**Affiliations:** 1 School of Agriculture and Food, Faculty of Veterinary and Agricultural Sciences, The University of Melbourne, Parkville, VIC 3010 Australia; 2 School of BioSciences, Faculty of Science, The University of Melbourne, Parkville, VIC 3010 Australia; 3 Melbourne Veterinary School, Faculty of Veterinary and Agricultural Sciences, The University of Melbourne, Werribee, VIC 3030 Australia; 4 Rivalea Australia Pty Ltd, Corowa, NSW 2646 Australia; 5 Centre for Muscle Research, Department of Anatomy and Physiology, The University of Melbourne, Parkville, 3010 Australia; 6 Department of Animal Science, Cornell University, Ithaca, NY 14853-4801 USA; 7 Faculty of Biological Sciences, The University of Leeds, Leeds, LS2 9JT UK

**Keywords:** Adipogenesis, Angiogenesis, Fetal pig, Gestation, Heat stress, Sexual dimorphism, Skeletal muscle, Sows

## Abstract

**Background:**

There is evidence that sow heat stress (HS) during gestation affects fetal development with implications for impaired muscle growth. We have previously demonstrated that maternal HS during early to mid-gestation compromised muscle fibre hyperplasia in developing fetal pigs. Thus, we hypothesised these phenotypic changes are associated with a change in expression of genes regulating fetal skeletal muscle development and metabolism. To test this, at d 60 of gestation, RNA sequencing and immunohistochemistry were performed on fetal *longissimus dorsi* (LD) muscle biopsies collected from pregnant gilts that had experienced either thermoneutral control (CON, 20 °C, *n* = 7 gilts, 18 LD samples) or controlled HS (cyclic 28 to 33 °C, *n* = 8 gilts, 23 LD samples) conditions for 3 weeks.

**Results:**

A total of 282 genes were differentially expressed between the HS and CON groups in female LD muscles (false discovery rate (FDR) ≤ 0.05), whereas no differentially expressed genes were detected in male LD muscles between the two groups (FDR > 0.05). Gestational HS increased the expression of genes associated with transcription corepressor activity, adipogenesis cascades*,* negative regulation of angiogenesis and pro-inflammatory signalling in female LD muscles. Immunohistochemical analyses revealed a decreased muscle vascularity density in fetuses from HS group for both sexes compared to those from the CON group (*P* = 0.004).

**Conclusions:**

These results reveal gilt HS during early to mid-gestation altered gene expression profiles in fetal LD muscles in a sexually dimorphic manner. The molecular responses, including transcription and angiogenesis repressions and enhanced adipogenesis cascades, were exclusively observed in females. However, the associated reductions in muscle vascularity were observed independently of sexes. Collectively this may indicate female fetal pigs are more adaptive to gestational HS in terms of gene expression changes, and/or there may be sexually dimorphic differences with respect to the timing of muscle molecular responses to gestational HS.

**Supplementary Information:**

The online version contains supplementary material available at 10.1186/s40104-022-00730-2.

## Background

Livestock species, such as the pig, are sensitive to elevated ambient temperatures, hence climate change presents a challenge to the pig industry through an increased incidence of heat stress (HS). Heat stress arises when animals are unable to maintain their core body temperatures within normal homeothermic limits, leading to an increase in thermoregulatory responses that compromise productivity [1]. Due to the critical role of skeletal muscles in regulating energy metabolism [2], they are likely to be one of the main targets for HS. In pigs, skeletal muscles represent a large proportion of the body mass and are one of the most important economic traits. Heat stress can result in muscle oxidative stress [3, 4], inflammation [5] and genome-wide epigenetic modifications that modulate stress responses and energy metabolism in pig skeletal muscles [6]. Increased temperatures have been observed to reduce ribosomal biogenesis and elicit an increased heat shock protein response in cultured porcine satellite cells [7]. These results show that pig skeletal muscles are sensitive to thermal stress, and when combined with reductions in feed intake and increased thermoregulatory efforts the overall impacts can compromise pig production efficiency [1].

In addition to the direct impact of postnatal HS on pig skeletal muscles, there is growing evidence that skeletal muscle growth of pig progeny can be affected by sow heat exposure during gestation [8]. Controlled climate experiments have shown that gestational HS causes lasting effects on the progeny, with reductions in muscle accretion and increased adiposity observed at the finishing stage [9]. Furthermore, sow HS during the first half of gestation contributed to increased subcutaneous fat thickness [10] and decreased loin muscle area in the offspring [11]. A recent study showed that piglets born to sows mated and gestated during summer were lighter at birth, while the carcasses of those born-light progeny tended to deposit less lean and more fat tissue in the loin [12]. Although the exact mechanisms have yet to be fully explored, placental insufficiency and impaired fetal muscle development may be contributing to this phenotype as postnatal productivity of livestock can be programmed by prenatal life [13]. One of the primary thermoregulatory mechanisms of sows in response to HS might be a redistribution of blood flow to the periphery to augment radiant heat loss. This redistribution results in impaired blood flow to certain organs, including the placenta [14]. We have shown that maternal HS between d 40 and 60 of gestation, corresponding to a period of robust placental growth, reduces placental efficiency and fetal muscle fibre number density at mid-gestation [15]. In fetal pigs, early to mid-gestation is also characterised by the onset of fetal primary fibre myogenesis [16], making the developing fetus and the skeletal muscle in particular sensitive to maternal stressors during this critical stage. Therefore, in the current study we hypothesised that gilt HS during early to mid-gestation altered gene expression profiles underpinning skeletal muscle development and metabolism in the developing fetus.

## Methods

### Animal ethics

The animal procedures were reviewed and approved by the Animal Ethics Committee of The University of Melbourne (Ethics Id: 1714365.2). The experimental protocols followed the Australian Code for the Care and Use of Animals for Scientific Purposes (8th edition; National Health and Medical Research Council, 2013).

### Animals and experimental design

Fetal *longissimus dorsi* (LD) muscle samples were obtained from animals used in a previously published study [15]. Briefly, fifteen primiparous sows (gilts) (Large White × Landrace) were sourced from a commercial farm (Huntly Piggery, Victoria, Australia). The gilts were artificially inseminated (d 0) using semen collected from a single sire followed by pregnancy confirmation on d 28 of pregnancy. The pregnant gilts were then transported to the climate-controlled facility, University of Melbourne, and individually housed in floor pens (220 cm × 120 cm) for acclimation. The climate conditions set for acclimation were constant 20 °C and ~ 50% relative humidity. At d 40 of gestation, the pregnant gilts were assigned to either the thermoneutral control group (CON; *n* = 7; constant 20 °C; ~ 50% relative humidity) or the cyclic heat stress group (HS; *n* = 8; 33 °C between 09:00 and 17:00 h and 28 °C between 17:00 and 9:00 h; ~ 50% relative humidity) and housed in the respective conditions for 3 weeks until d 60 of gestation. The pregnant gilts were fed twice daily with a commercially available gestational diet at 2.0 kg per day (equivalent to 1.3 × metabolizable energy (ME)) as per commercial procedures. Thus, all pregnant gilts had equivalent nutrient intakes as well as ad libitum access to water throughout the experimental period.

### Tissue collection and morphometric analysis

At the end of the climate-controlled period at d 60 of gestation, the pregnant gilts were sedated and humanely euthanised via intracardiac injections of Lethabarb (pentobarbitone sodium; 162.5 mg/kg liveweight; Virbac Animal Health, NSW, Australia) followed by collection of their uteri. In each gilt (litter), one uterine horn was randomly chosen from which a total of three developing fetuses located at the cervical, middle and distal uterine position, respectively, were obtained (*n* = 45 fetuses). The fetuses were sexed and a biopsy of LD muscle was collected from each fetus and snap frozen in liquid nitrogen. The frozen muscle samples were pulverised by a tissue pulverizer under liquid nitrogen and stored at − 80 °C until further processing.

### Total RNA extraction, cDNA library preparation and sequencing

Muscle total RNA was extracted using ReliaPrep RNA tissue Miniprep System (Cat# Z6111; Promega, Madison, WI, USA) as per the manufacturer’s instructions. RNA integrity and concentration were assessed using the RNA Nano 6000 Assay Kit of the Bioanalyzer 2100 system (Agilent Technologies, CA, USA) according to the manufacturer’s instructions. Total RNA purity was verified using a nanodrop spectrophotometer (Thermo Scientific, Walton, MA, USA). The muscle samples used for downstream processing had an average RNA integrity number (RIN) of 9.57 ± 0.18 (mean ± SD) and a 28/18S ratio of 1.65 ± 0.09 (mean ± SD). Four samples (from two females and two males) were excluded from the experiment due to poor RNA quality. The sample distributions were as follows: 10 female and 8 male muscle samples in the CON group; and 17 female and 6 male muscle samples in the HS group. The sequencing library was prepared using the NEBNext® Ultra™ Directional RNA Library Prep Kit for Illumina® (New England Biolabs, Ipswich, MA, USA) as per the manufacturer’s instructions. The cDNA library was sequenced on an Illumina NovaSeq6000 sequencing platform (Annoroad Gene Technology Corporation, Beijing, China), and 150 base-pair, paired-end reads were generated from each sample.

### Sequencing data processing

The sequencing data processing followed the pipeline as previously described [17]. The quality of sequencing reads was verified using FastQC software version 0.11.9 [18]. The Illumina adaptors and low-quality bases were removed before further processing. The clean reads for downstream processing and analyses had a Phred score above 30, equivalent to a nucleobase accuracy above 99.9%. The pig reference genome (*sus scrofa* 11.1) DNA sequence (FASTA) and the annotation GTF files were obtained from Ensembl database. Clean reads were mapped against the pig reference genome using Hisat2 version 2.1.0 [19]. The samples had an average alignment rate (%) of 96.9 ± 0.3 (mean ± SD). The aligned reads were counted per gene using HTSeq software (version 0.11.3) with reverse strand interpretation [20].

### Differential gene expression, and gene ontology (GO) and pathway enrichment analyses

Differential gene expression analyses were performed in edgeR version 3.30.0 [21] in R software version 4.0.0 [22]. Genes with low read counts were filtered and library sizes were normalised using the trimmed mean of M-values (TMM) method [23]. Gestational temperature treatment and fetal sex were included in the model. Fetuses within the same litter was adjusted for repeated measures. Differential expression analyses were performed for six pairs of comparisons within the model (HS vs. CON across males and females; HS vs. CON in females; HS vs. CON in males; males vs. females across HS and CON; males vs. females in HS; and males vs. females in CON). To control false positive findings, the Benjamini-Hochberg false discovery rate (FDR) correction was performed. Genes with an adjusted *P* value (FDR) ≤ 0.05 were considered as differentially expressed for each pairwise comparison. Gene ontology statistical overrepresentation tests were performed against the up- and downregulated genes separately using g:Profiler annotated for pigs [24]. Gene set enrichment analysis [25] was performed in fgsea R package version 1.14.0 [26]. Briefly, the complete gene list (13,267 genes) obtained from edgeR [21] was ranked based on log_2_ fold change (FC) in an ascending order (the most upregulated gene to the most downregulated gene by fold change), and the rank metric for each gene was calculated as signed (log_2_ (FC)) × − log_10_ (*P* value). The pre-ranked gene list was analysed against the Kyoto Encyclopedia of Genes and Genomes (KEGG) and REACTOME gene sets (C2), and transcription factor targets (C3) downloaded from the Molecular Signatures Database (MSigDB) v7.2 [25]. Gene ontology or pathway terms with an adjusted *P* value (FDR) ≤ 0.05 were considered enriched or overrepresented.

### Immunohistochemistry

To further quantify the impact of gestational HS on fetal muscle vascularity, immunohistochemistry (IHC) was performed to test the differences in LD muscle blood vessel density by quantifying the relative abundance of the platelet endothelial cell adhesion molecule (CD31), a biomarker of vascular endothelial cells [27]. *Longissimus dorsi* samples were obtained from two fetuses per litter (CON: *n* = 14 LD samples (7 males and 7 females), HS: *n* = 16 LD samples (7 males and 9 females). The LD samples were fixed and paraffin-embedded as per methods previously described [15]. The embedded tissue cross-sectional areas were cut at a thickness of 5 μm by a rotary microtome (Leica biosystems, Victoria, Australia). The sections were fully dried at 37 °C overnight and de-paraffinised using xylene solutions. The sections were rehydrated through graded ethanol solutions (100%, 90% and 70%) for 2 min each. Heat-mediated antigen retrieval was performed using citrate buffer (pH = 6). The subsequent IHC experiment was performed using the Novolink Max Plymer Detection System (Cat# RE7280-K, Leica biosystems, Victoria, Australia) as per the manufacturer’s instructions. The sections were incubated with anti-CD31 rabbit primary antibody (1:100 dilution, Cat# ab28364, Abcam, Cambridge, MA, USA) diluted in IHC diluent (Cat# RE7133-CE, Leica, Nussloch, Germany) overnight at 4 °C. This primary antibody was validated for pig tissue IHC, as previously described [28]. Finally, the sections were counterstained with hematoxylin and dehydrated with 100% ethanol and xylene solutions. Images were taken using a microscope (Olympus, Tokyo, Japan) at 100× total magnification. For each section (sample), 10 microscopic fields were randomly chosen and analysed. The positive CD31 staining areas were identified and calculated using ImageJ software (NIH, USA). The averaged blood vessel density across the 10 microscopic fields was calculated and expressed as area percentages of positive CD31 staining per square micrometre of the microscopic section.

### Statistical analyses for the immunohistochemistry data

Data normality was verified by Shapiro–Wilk’s test before they were analysed by linear mixed models (REML) (Genstat 18th ed., VSN International, Hemel Hempstead, UK). The fixed terms were gestational temperature treatment, fetal sex and their interactions with dam ID used as a random factor. A *P* value ≤ 0.05 was considered significantly different.

## Results

### Gilt thermal stress and fetal morphology

The impacts of gestational HS on gilt thermal stress responses, and placental and fetal morphology have been reported elsewhere [15]. Those data were reproduced here in order to relate them to the transcriptome data generated from the current study. Gilt averaged respiration rates (CON = 23 ± 4 breaths per min vs. HS = 101 ± 4 breaths per min, *P* < 0.001), skin temperatures (CON = 30.3 ± 0.2 °C vs. HS = 36.6 ± 0.2 °C, *P* < 0.001) and rectal temperatures (CON = 37.5 ± 0.08 °C vs. HS = 38.3 ± 0.07 °C, *P* < 0.001) were increased by heat exposure. Individual fetal weight was not affected by HS at fetal d 60 of age (*P* > 0.05), but males were heavier than females (male = 96.3 ± 3.7 g vs. female = 91.4 ± 3.7 g, *P* = 0.019). Individual placental weight was increased by gestational HS (CON = 90 ± 8 g vs. HS = 110 ± 7 g, *P* = 0.041). There was a main effect of gestational temperature on fetal:placental weight ratio (CON = 1.16 ± 0.08 vs. HS = 0.91 ± 0.07, *P* = 0.013). A trend for an interaction between gestational temperature and fetal sex was observed suggesting that reduction in fetal:placental weight ratio by HS exposure in utero was most pronounced in female fetuses (*P* = 0.081). Fetal muscle fibre number density (CON = 858 ± 41 vs. HS = 722 ± 38 fibres per mm^2^ area of field, *P* = 0.032) and nuclei number density (CON = 1680 ± 40 vs. HS = 1423 ± 38 nuclei per mm^2^ area of field, *P* < 0.001) were reduced in fetuses from the HS group. No interaction effect was observed between gestational temperature treatment and gender for the measured muscle parameters (*P* > 0.1).

### Differentially expressed genes and gene ontology and pathway enrichment analyses

**Fig. 1:**
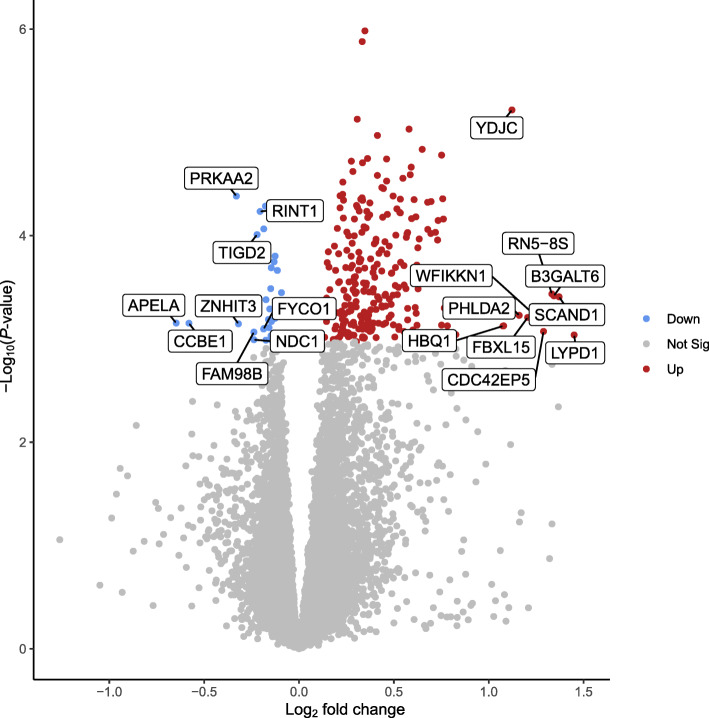
Volcano plot of the differentially expressed genes (DEG) for the comparison of heat stress (HS, *n* =17) vs. control (CON, *n* = 10) in female fetal longissimus dorsi (LD) muscles at fetal d 60 age. Blue, red and grey dots denote downregulated (FDR (false discovery rate) ≤ 0.05), upregulated (FDR ≤ 0.05) and nondifferentially expressed (FDR > 0.05) genes in the HS group, respectively, compared to the CON group. The most up- and down-regulated genes by fold change are labelled

A total of 31,907 genes were annotated in pig fetal LD muscles, of which 13,267 genes were expressed at fetal d 60 age after removing low count genes. There were 282 differentially expressed genes (DEG) between female LD muscles in the HS and CON groups (FDR ≤ 0.05, Fig. 1), whereas no DEG was detected in male LD muscles (FDR > 0.05, Table 1). Among the 282 genes expressed at different levels in the HS vs. CON groups in females, 29 genes were downregulated, and 253 genes were upregulated by gestational HS. Table 2 lists a subset of the DEG for the comparison of HS vs. CON in female LD muscles, including the top five most up- and downregulated genes by fold change, and genes involved in transcription, angiogenesis, fibrogenesis and metabolism that are a particular focus herein. When combining females and males, no DEG was detected between HS and CON groups (FDR > 0.05). A total of 13 genes (12 of which were linked to sex chromosomes) were differentially expressed (FDR ≤ 0.05) between LD muscles in females and males, regardless of gestational temperature treatment. A total of 16 genes (13 of which linked to sex chromosomes) were differentially expressed between females and males in the CON group, and 13 genes (11 of which linked to sex chromosomes) were differentially expressed between females and males in the HS group (Table 1). The full list of the DEG for each pair of comparison can be accessed via Additional file 1: Table S1.

**Table 1:** The number of differentially expressed genes in each pair of comparison

Pairs of comparison	Numbers of differentially expressed genes	Numbers of up- and downregulated genes^a^
HS vs. CON across all sexes	0	–
HS vs. CON in females	282	253 upregulated; 29 downregulated
HS vs. CON in males	0	–
Males vs. Females across CON and HS	13	1 upregulated; 12 downregulated
Males vs. Females in CON	16	2 upregulated; 14 downregulated
Males vs. Females in HS	13	2 upregulated; 11 downregulated

^a^The up- and downregulated genes are expressed as heat stress (HS) relative to control (CON) group and males relative to females in their respective comparisons

**Table 2:** A subset of differentially expressed genes that includes the top five most up- and downregulated genes by fold change, and genes involved in transcription, angiogenesis, fibrogenesis and metabolism that are a particular focus herein for the comparison of heat stress (HS, *n* = 17) vs. control (CON, *n* = 10) groups in female fetal *longissimus dorsi* (LD) muscles at fetal d 60 age

Gene symbol	Description	log_**2**_ fold change	False discovery rate
*NCAM2*	Neural cell adhesion molecule 2	−0.75	0.038
*APELA*	Apelin receptor early endogenous ligand	−0.65	0.042
*CCBE1*	Collagen and calcium binding EGF domains 1	−0.58	0.042
*PRKAA2*	Protein kinase AMP-activated catalytic subunit alpha 2	−0.33	0.016
*ZNHIT3*	Zinc finger HIT-type containing 3	−0.32	0.043
*ACACA*	Acetyl-CoA carboxylase alpha	−0.16	0.042
*NR3C1*	Nuclear receptor subfamily 3 group C member 1	−0.13	0.024
*MAPK1*	Mitogen-activated protein kinase 1	−0.09	0.031
*COL4A2*	Collagen type IV alpha 2 chain	0.18	0.037
*PXDN*	Peroxidasin	0.23	0.016
*COL6A2*	Collagen type VI alpha 2 chain	0.25	0.024
*NCOR1*	Nuclear receptor corepressor 1	0.28	0.016
*DMAP1*	DNA methyltransferase 1 associated protein 1	0.29	0.047
*RXRA*	Retinoid X receptor alpha	0.29	0.016
*EPN2*	Epsin 2	0.36	0.042
*SREBF1*	Sterol regulatory element binding transcription factor 1	0.37	0.039
*IRAK1*	Interleukin 1 receptor associated kinase 1	0.37	0.045
*NOTCH1*	Notch receptor 1	0.37	0.016
*PPARGC1B*	PPARG coactivator 1 beta	0.38	0.045
*EID1*	EP300 interacting inhibitor of differentiation 1	0.42	0.022
*JAG2*	Jagged canonical Notch ligand 2	0.42	0.033
*TRAF2*	TNF receptor associated factor 2	0.46	0.032
*TOLLIP*	Toll interacting protein	0.50	0.043
*LAMA5*	Laminin subunit alpha 5	0.59	0.016
*SLC2A4RG*	SLC2A4 regulator	0.59	0.016
*EPN1*	Epsin 1	0.61	0.016
*COL18A1*	Collagen type XVIII alpha 1 chain	0.62	0.016
*KLF2*	Kruppel like factor 2	0.62	0.024
*CDC42EP5*	CDC42 effector protein 5	1.29	0.045
*RN5-8S*	5.8S ribosomal RNA	1.33	0.032
*B3GALT6*	Beta-1,3-galactosyltransferase 6	1.34	0.032
*SCAND1*	SCAN domain containing 1	1.37	0.032
*LYPD1*	LY6/PLAUR domain containing 1	1.45	0.047

### Gene ontology and pathway overrepresentation analyses

**Fig. 2:**
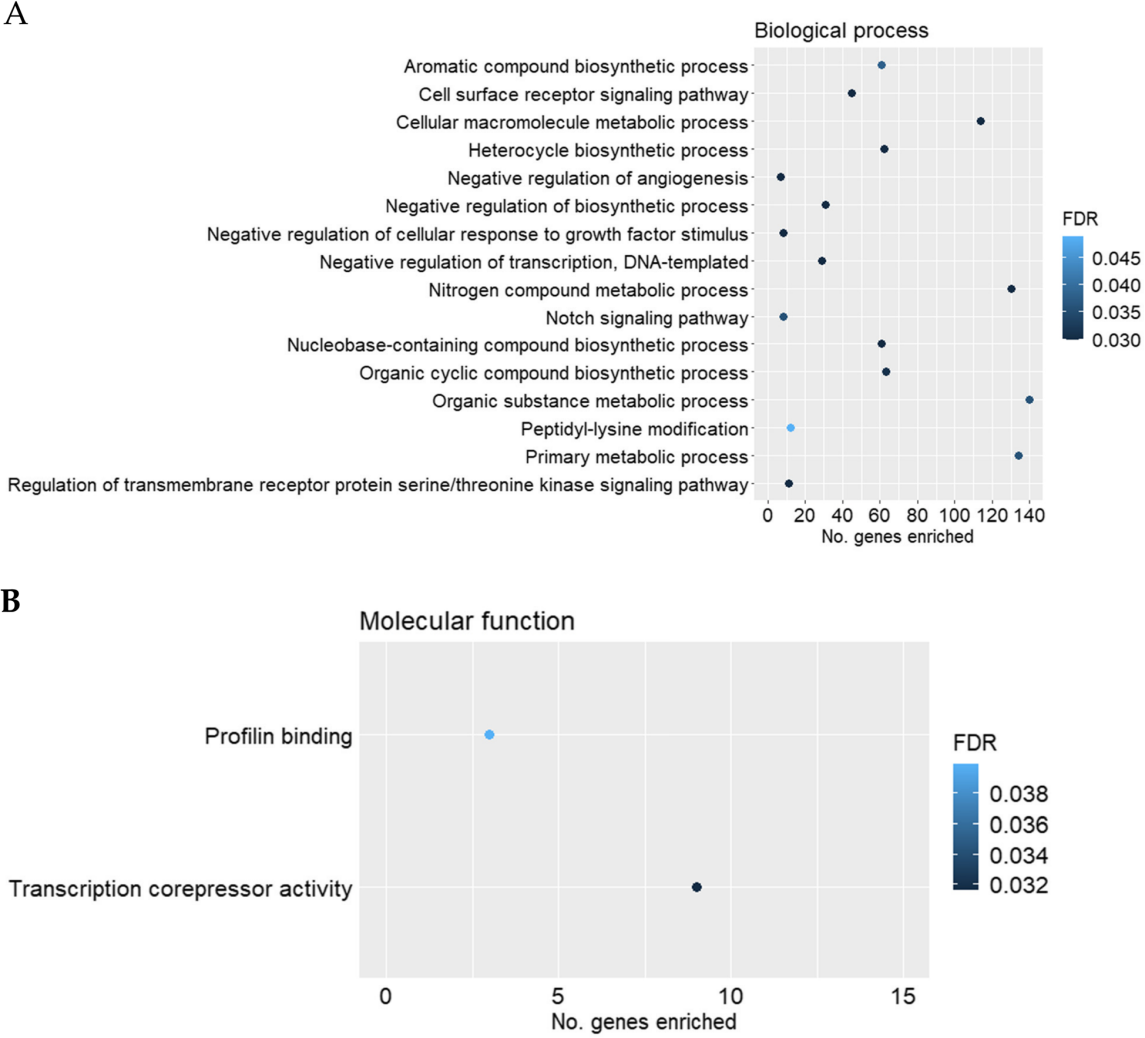
Scatterplots of gene ontology (GO) terms significantly enriched (FDR ≤ 0.05) by the upregulated genes for the comparison of heat stress (HS, *n* = 17) vs. control (CON, *n* = 10) in female fetal *longissimus dorsi* (LD) muscles at fetal d 60 age for the GO domains of **A** biological process, **B** molecular function

**Fig. 3:**
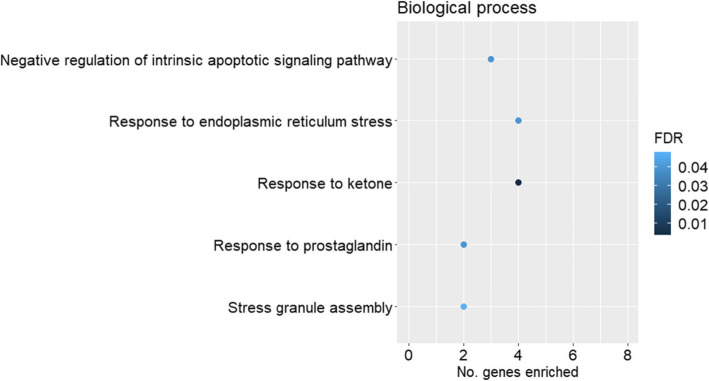
Scatterplots of gene ontology (GO: biological process) terms significantly enriched (FDR ≤ 0.05) by the downregulated genes for the comparison of heat stress (HS, *n* = 17) vs. control (CON, *n* = 10) in female fetal *longissimus dorsi* (LD) muscles at fetal d 60 age for the GO domain of biological process

Gene ontology overrepresentation analysis was performed for the up- and downregulated genes for each pairwise comparison against the GO domains of biological process and molecular function. Among the upregulated genes for the comparison of HS vs. CON in females, negative regulation of transcription - DNA templated (GO:0045892, FDR = 0.030), negative regulation of angiogenesis (GO:0016525, FDR = 0.030), Notch signalling pathway (GO:0007219, FDR = 0.036) and negative regulation of cellular response to growth factor stimulus (GO:0090288, FDR = 0.030) were among the enriched biological process GO terms (Fig. 2A). In the domain of molecular function (Fig. 2B), transcription corepressor activity (GO:0003714, FDR = 0.032) and profilin binding (GO:0005522, FDR = 0.040) were significantly enriched by the upregulated genes (HS compared to CON in females). Among the downregulated genes (HS compared to CON in females), response to ketone (GO:1901654, FDR = 0.004) and response to endoplasmic reticulum stress (GO:0034976; FDR = 0.040) were among the enriched GO terms in the domain of biological process (Fig. 3). None of the significantly downregulated genes (HS compared to CON in females) contributed to significantly enriched GO terms for the domain of molecular function (FDR > 0.05).

In the comparison between male and female fetuses, translation initiation (GO:0006413) was significantly enriched for the group of CON (FDR = 0.037), HS (FDR = 0.015), and CON and HS combined (FDR = 0.024). In addition, X-linked dominant inheritance (HP:0001423) was enriched for the group of CON (FDR = 0.011), HS (FDR = 0.011), and CON and HS combined (FDR = 0.031). The full list of significantly enriched gene ontology terms, pathways, FDRs and their associated genes can be accessed via Additional file 2: Table S2.

### Gene set enrichment analyses (GSEA)

The comparison of HS vs. CON in females was chosen for GSEA as it was the primary focus herein. Gene set enrichment analyses identified that a total of nine pathways, including signalling by insulin receptor (FDR = 0.049) and MAPK family signalling cascades (FDR = 0.049), were significantly enriched in the REACTOME database for the comparison of HS vs. CON in females (Table 3). In addition, the GO term assembly of collagen fibrils and other multimeric structures showed a trend toward enrichment by the upregulated genes (FDR = 0.08; Additional file 3: Table S3). DNA replication (FDR = 0.018), adherens junction (FDR = 0.013) and JAK-STAT signalling pathway (FDR = 0.005) were among pathways significantly enriched (FDR < 0.05) in the KEGG (Kyoto Encyclopedia of Genes and Genomes) database (Table 4). A total of 33 transcription factor binding motifs were significantly enriched for the comparison of HS vs. CON in females (Table 5). A full list of the GSEA results, including the associated genes, can be accessed in Additional file 3: Table S3.

**Table 3:** Reactome pathways significantly enriched (false discovery rate (FDR) ≤ 0.05) by the ranked gene list for the comparison of heat stress (HS) vs. control (CON) in female fetal *longissimus dorsi* (LD) muscles at fetal d 60 age

Reactome pathway	False discovery rate	NES
Interleukin-3, Interleukin-5 and GM-CSF signaling	0.006	−2.09
Calnexin/calreticulin cycle	0.029	−1.96
N-glycan trimming in the ER and calnexin/calreticulin cycle	0.049	−1.89
MAPK family signaling cascades	0.049	−1.45
Fc epsilon receptor (FCERI) signaling	0.049	−1.64
Signaling by insulin receptor	0.049	−1.81
Signaling by receptor tyrosine kinases	0.049	−1.37
FLT3 Signaling	0.049	−1.50
Resolution of Abasic Sites (AP sites)	0.049	1.82

*NES* Normalised enrichment score. A positive NES, the pathway was mostly enriched by upregulated genes (HS compared to CON). A negative NES, the pathway was mostly enriched by the downregulated genes (HS compared to CON)

**Table 4:** KEGG pathways significantly enriched (false discovery rate (FDR) ≤ 0.05) by the ranked gene list for the comparison of heat stress (HS) vs. control (CON) in female fetal *longissimus dorsi* (LD) muscles at fetal d 60 age

KEGG pathway	False discovery rate	NES
JAK/STAT signaling pathway	0.005	− 1.90
Adherens junction	0.013	−1.80
DNA replication	0.018	1.84
Epithelial cell signaling in *Helicobacter pylori* infection	0.026	−1.81

*NES* Normalised enrichment score. A positive NES, the pathway was mostly enriched by upregulated genes (HS compared to CON). A negative NES, the pathway was mostly enriched by the downregulated genes (HS compared to CON)

**Table 5:** Transcription factor prediction gene sets significantly enriched (false discovery rate (FDR) ≤ 0.05) by the ranked gene list for the comparison of heat stress (HS) vs. control (CON) in female fetal l*ongissimus dorsi* (LD) muscles at fetal d 60 age

Transcription factor targets	False discovery rate	NES
YNGTTNNNATT_unknown	0.000	−1.69
PGM3 target genes	0.001	1.81
PCGF1 target genes	0.001	1.60
TCCATTKW_ unknown	0.001	−1.73
ZNF436 target genes	0.001	1.67
ATF5 target genes	0.002	−1.55
CHAMP1 target genes	0.002	−1.78
FOXG1 target genes	0.002	−1.48
F10 target genes	0.002	−1.66
HSD17B8 target genes	0.002	1.54
RGAANNTTC_HSF1_01	0.012	−1.47
MEF2_Q6_01	0.014	−1.53
HSF1_01	0.016	−1.56
MYB_Q5_01	0.016	−1.54
TGACATY_ unknown	0.017	−1.34
YKACATTT_ unknown	0.019	−1.49
HES4 target genes	0.019	−1.38
IGLV5_37 target genes	0.024	1.47
H1_6 target genes	0.025	1.47
SMTTTTGT_ unknown	0.026	−1.41
DLX2 target genes	0.026	−1.51
MSX1 target genes	0.032	−1.52
NFMUE1_Q6	0.038	−1.44
EMX1 target genes	0.038	−1.52
PR_02	0.039	−1.65
GCCATNTTG_YY1_Q6	0.039	−1.35
HDAC4 target genes	0.041	1.40
SOX5_01	0.044	−1.47
NRF2_01	0.044	−1.43
TCCCRNNRTGC_ unknown	0.045	−1.46
AAAYRNCTG_ unknown	0.045	−1.36
TERF1 target genes	0.045	−1.44
STAT5A_04	0.050	−1.47

### Immunohistochemistry

**Fig. 4:**
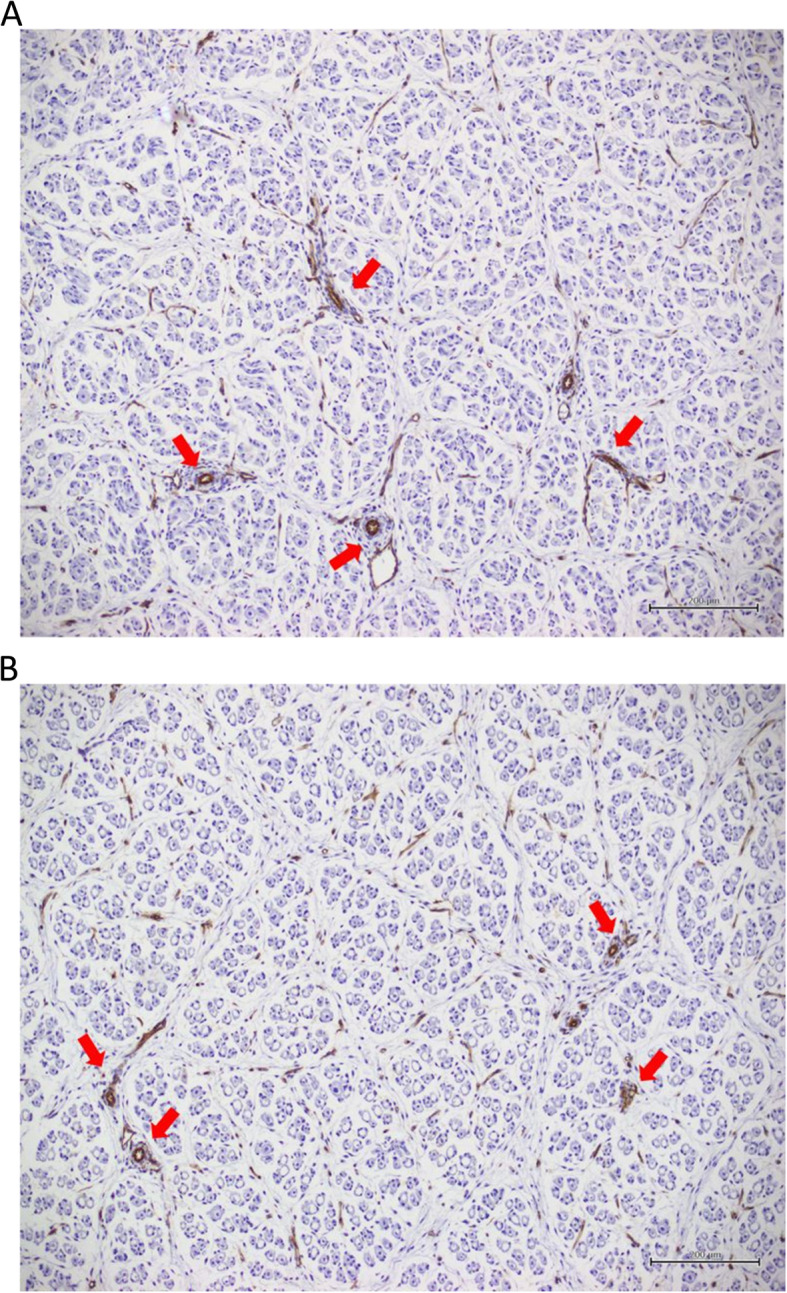
Photomicrographs of representative positive CD31 staining, the biomarker for blood vessel endothelium (examples indicated by red arrows), in fetal *longissimus dorsi* (LD) muscles of fetuses from the control (CON; A) or heat stress (HS; B) groups. CON: *n* = 14 LD samples, HS: *n* = 16 LD samples. Total magnification: 100×. Bars: 200 μm

Fetal LD muscles from pregnant gilts experiencing HS had decreased area density for positive CD31 staining compared to their counterparts from CON gilts (HS = 0.52 ± 0.03% vs. CON = 0.68 ± 0.03%, *P* = 0.004, Fig. 4). Female and male fetuses had a similar positive CD31 staining area density in LD muscles (Female = 0.58 ± 0.02% vs. Male = 0.62 ± 0.03%, *P* > 0.1). The interaction between gestational temperature treatment and fetal sex for positive CD31 staining area density was not significant (*P* > 0.1).

## Discussion

The principal findings of this study were that when pregnant gilts experienced HS during early to mid-gestation alterations in fetal skeletal muscle gene expression were exclusively in females. The molecular responses caused by gestational HS in the female fetus were associated with transcription repression, enhanced adipogenesis and fibrogenesis, while angiogenesis was downregulated. Moreover, muscle blood vessel density was reduced by gestational HS independent of fetal sexes. These data indicate that fetal LD muscles are susceptible to maternal HS during gestation and provide a novel explanation for the change in skeletal muscle phenotypes of the progeny born to heat-stressed sows.

An interesting finding of the present study was that fetal LD muscles showed a sex-specific response to gestational HS at the gene expression level, such that females, but not their male littermates, displayed the adaptive changes at mid-gestation when the gilts were exposed to HS. The reason for this sexually dimorphic molecular response to gestational HS is not clear. However, these findings can be potentially explained by temporal differences in the timing of muscle development and responses to prenatal stress between sexes. This is reflected by findings from the current study that males were heavier than females at the similar fetal age. Others reported that skeletal muscles of male fetuses develop greater than those of female fetuses in cattle [29], although this in utero sexual dimorphic effect remains to be verified in pigs. Postnatally though the semitendinosus muscles in female grower pigs are more susceptible to HS-induced mitochondrial damage than those in male pigs [30]. Thus, the absence of gene expression changes in male muscles at mid-gestation might be due to their earlier adaptation to maternal HS, as a consequence of rapid muscle growth, hence the lack of differences at the time of sampling on d 60. Changes in fetal skeletal muscle characteristics are likely underpinned by placental insufficiency [31]. We have previously demonstrated that placentae from the female fetuses exhibited a sex-specific reduction in placental efficiency (fetal/placental weight) in response to maternal HS at mid-gestation, and the placental insufficiency of those female placentae was associated with impaired nutrient transport capacity [15, 17]. The current data therefore reinforce and extend our previous findings by demonstrating that HS-induced placental insufficiency can be associated with altered skeletal muscle molecular responses in the fetus.

Notably, female LD muscles from heat-stressed pregnant gilts showed a lower capacity for gene transcription. This was evidenced by an upregulation of genes associated with transcription corepressor activity by gestational HS, suggesting transcription repression. Skeletal muscle development occurs via a balance between transcription activation and repression, and a defect in gene transcription implies developmental abnormalities [32]. We have identified transcription corepressors, including metastasis associated 1 (*MTA1*), nuclear receptor corepressor (*NCOR1*), DNA methyltransferase 1 associated protein 1 (*DMAP1*), C-terminal binding protein 1 (*CTBP1*) and E1A-like inhibitor of differentiation-1 (*EID1*) among the upregulated gene lists. For example, overexpression of *NCOR1* inhibits muscle cell myogenesis through directly inhibiting the activity of MyoD, the master regulator of muscle myogenesis [33]. Similarly, overexpression of EID1 can inhibit skeletal muscle gene transcription by competitively binding with MyoD coactivators in skeletal muscles [34]. A possible explanation for the transcription repression might be DNA methylation-induced silencing of gene expression. This is supported by the overexpression of *DMAP1*, an activator of DNA methylation [35], in the HS group. The inhibition of transcription also suggests a lower accumulation of myonuclei in skeletal muscles, potentially affecting later muscle hypertrophy [36]. These findings are also supported by our previous findings, showing myonuclei numbers were lower in fetal muscles from heat-stressed pregnant gilts [15]. Collectively, these data show that gestational HS reduced gene expression potential in female fetuses, possibly programming cellular phenotypes. This may be associated with epigenetic modifications, and further studies are warranted to test the change in genome-wide DNA methylation in progeny skeletal muscles, due to maternal HS.

The present data showed that gestational HS inhibited the formation of new blood vessels in female LD muscles evidenced by a change in expression of genes that inhibited angiogenesis, while the endothelium abundance and vascularity were globally reduced in both sexes. The sex-specific change in gene expression might be related to timing of muscle adaptation between sexes as aforementioned. Furthermore, caution should be taken for reconciling the morphological and gene expression changes, because angiogenesis involves the formation of new capillaries continuing on from existing blood vessels, whereas endothelial cells line the interior of each individual blood vessel, hence are a proxy for the total area occupied by blood vessels. Therefore, these two measurements may represent different blood vessel characteristics and are not necessarily directly related or could be temporarily distinct events. For example, the initial response of LD muscles to gestational HS could be to decrease the density of blood vessels in both sexes, whilst changes in angiogenesis may be evident at d 60 only in female fetuses possibly due to greater placental insufficiency. This can be potentially explained by a lack of oestrogen (known to drive angiogenesis [37]) in female fetuses from the HS group, whilst testosterone production, which has a role in sustaining angiogenesis [38] similar to those of control fetuses, is elevated between d 40 to 60 of gestation in male fetal pigs [39]. The current study design does not provide insights explaining the dichotomy between the female and male angiogenic gene expression relative to endothelial cell density in response to gestational HS on fetal LD muscles. Nevertheless, impaired angiogenesis and vascularity can further influence muscle nutrient uptake, oxidative function and muscle fibre development at later stages.

An important finding of this study was that gene expression of transcription factors involved in adipogenesis cascades was upregulated in female skeletal muscles from the HS group. Of note, fetal muscle expression of peroxisome proliferator-activated receptor (PPAR) gamma coactivator 1-beta (*PPARGC1B/PGC-1β*) was increased by gestational HS. The *PPARGC1B* regulates cell mitochondrial biogenesis, fatty acid oxidation and coordinates lipogenesis [40]. In addition, the gene product of *PPARGC1B* can bind and activate the function of PPARG, the essential transcription factor for adipogenesis [41]. Increased *PPARGC1B/PGC-1β* expression can stimulate lipogenic gene expression and contribute to adipocyte differentiation [42, 43]. The PPARG mediates its function via heterodimerisation with the retinoid X receptor alpha (*RXRα*), as PPARs are ligand-activated transcription factors [44]. In the present study, *RXRα* gene expression was also increased in female LD muscles from the HS group, reinforcing the upregulation of the PPARG/RXRα signalling. Furthermore, we also identified higher expression of the sterol regulatory element-binding transcription factor 1 (*SREBF1*), also known as adipocyte determination and differentiation-dependent factor 1 (*ADD1*), in the female LD muscles from the HS group. The *SREBF1/ADD1* is a potent activator of PPARG/RXRα activity during adipocyte differentiation [45], possibly via the coactivation of *PPARGC1B* [43]. These data are consistent, in part, with another study showing increased gene expression of adipogenic transcription factors, including *PPARG* and *SREBP1C*, in adipose tissues of neonatal piglets born to sows experiencing HS during late gestation and lactation [46]. Taken together, these data demonstrated the change in expression of genes associated with enhanced adipogenesis cascades and altered energy metabolism in fetal LD muscles by gestational HS. Further studies need to investigate whether gene expression changes translate to protein expression in fetal skeletal muscles. Although fat deposition in fetal/newborn piglets is relatively low, it can be detected in fetal skeletal muscles at mid-gestation [47]. Upregulation of adipogenic transcription factors at early developmental stages potentially programs fetal muscle growth and affects carcass traits of the offspring born to sows experiencing HS during early-mid gestation [10–12].

In addition to upregulation of adipogenic transcription factors, the current study identified increased expression of genes associated with collagen formation by gestational HS in the female fetuses, suggesting increased levels of fibrogenesis and connective tissue deposition. In particular, the genes *COL4A2* and *LAMA5* encode subtypes of collagen IV and laminin, respectively, which are involved in the formation of basal lamina/endomysium surrounding individual muscle fibres [48]. Moreover, the gene product of *PXDN*, peroxidasin, mediates extracellular matrix formation and tissue fibrogenesis in myofibroblasts [49]. These data imply an accumulation of endomysial connective tissues in LD muscles of female fetuses from the heat-stressed gilts. Although collagen and extracellular matrix are required to scaffold muscle fibres, interestingly, increased levels of connective tissues and fat were observed in skeletal muscles from smaller fetal pigs compared to their larger littermates [47], suggesting negative roles of increased intramuscular fat and collagen on fetal muscle development. As myocytes and adipocytes are derived from common mesenchymal stem cells [50], myogenic and adipogenic cell lineages are reported as negatively coordinated [51]. Thus, the identified upregulation in gene expression of adipogenic and fibrogenic transcription factors indicates that myogenesis potential is less in those female fetal muscles by gestational HS. Future research is needed to investigate whether gestational HS shifts fetal skeletal muscles from myogenesis to fibrogenesis and adipogenesis.

The altered muscle metabolism in fetal skeletal muscles in females might be explained by fetal inflammation [52]. In this study, we found that gestational HS induced low-grade pro-inflammatory responses in LD muscles, evidenced by increased gene expression of pro-inflammatory cytokines, including tumor necrosis factor (TNF) receptor associated factor 2 (*TRAF2*), interleukin-1 receptor activated kinase 1 (*IRAK1*) and toll interacting protein (*TOLLIP*). For example, *TRAF2* is involved in TNF receptor signalling and NFkB activation, a major pathway involved in inflammatory signalling [53], while *IRAK1* and *TOLLIP* play a critical role in signalling transduction of interleukin-1 receptors (IL-1Rs) and toll-like receptors during inflammatory regulation [54, 55]. The increased inflammatory responses of the fetuses might be a consequence of an altered fetal immune system due to HS exposure in utero [56].

## Conclusions

The present study provides evidence that gestational HS during early to mid-gestation alters gene expression profiles of LD muscles at mid-gestation, in female but not male fetal pigs. These data suggest female fetuses might be more adaptive, and/or there may be sexually dimorphic differences in the timing of molecular responses to gestational HS in fetal skeletal muscles. Nevertheless, gestational HS suppressed fetal LD muscle capacity for gene transcription and angiogenesis in female fetuses. Fetal muscle blood vessel density was reduced by gestational HS in both sexes. Gilt HS during gestation upregulated the expression of genes associated with fetal LD muscle adipogenesis cascades and connective tissue formation, suggesting a negative impact on skeletal muscle myogenesis. These changes might be associated with indirect evidence of low-grade inflammation. Collectively, this may have implications for later fetal and postnatal muscle growth trajectory. Future studies specifically investigating the relevant protein expression changes are necessary to provide further insights into the functional impacts of our findings.

## Supplementary Information

**Additional file 1: Table S1.** Full list of the DEG for each pair of comparison.

**Additional file 2: Table S2.** Full list of significantly enriched gene ontology terms.

**Additional file 3: Table S3.** Full list of the GSEA results.

## Data Availability

The datasets used and/or analysed during the current study are available from the corresponding author on reasonable request.

## Abbreviations

LD: *Longissimus dorsi*
HS: Heat stress
CON: Control
RNA-seq: RNA sequencing
FDR: False discovery rate
RIN: RNA integrity number
GO: Gene ontology
MSigDB: Molecular Signatures Database
GSEA: Gene set enrichment analyses
KEGG: Encyclopedia of Genes and Genomes
IHC: Immunohistochemistry
DEG: Differentially expressed genes
CD31: Platelet endothelial cell adhesion molecule
MTA1: Metastasis associated 1
NCOR1: Nuclear receptor corepressor 1
DMAP1: DNA methyltransferase 1 associated protein 1
CTBP1: C-terminal binding protein 1
EID1: E1A-like inhibitor of differentiation-1
PPARGC1B: Peroxisome proliferator-activated receptor (PPAR) gamma coactivator 1-beta
RXRα: Retinoid X receptor alpha
SREBF1: Sterol regulatory element-binding transcription factor 1
COL4A2: Collagen type IV alpha 2 chain
LAMA5: Laminin subunit alpha 5
PXDN: Peroxidasin
TRAF2: Tumor necrosis factor (TNF) receptor associated factor 2
IRAK1: Interleukin-1 receptor activated kinase 1
TOLLIP: Toll interacting protein
IL-1Rs: Interleukin-1 receptors

## References

[1] Renaudeau D, Gourdine J-L, St-Pierre N (2011). A meta-analysis of the effects of high ambient temperature on growth performance of growing-finishing pigs. J Anim Sci.

[2] Herpin P, Louveau I, Damon M, Le Dividich J, Burrin DG, Mersmann HJ (2005). Environmental and hormonal regulation of energy metabolism in early development of the pig. Biology of growing animals.

[3] Montilla SIR, Johnson TP, Pearce SC, Gardan-Salmon D, Gabler NK, Ross JW, Rhoads RP, Baumgard LH, Lonergan SM, Selsby JT (2014). Heat stress causes oxidative stress but not inflammatory signaling in porcine skeletal muscle. Temperature..

[4] Ganesan S, Brownstein AJ, Pearce SC, Hudson MB, Gabler NK, Baumgard LH, Rhoads RP, Selsby JT (2018). Prolonged environment-induced hyperthermia alters autophagy in oxidative skeletal muscle in Sus scrofa. J Therm Biol.

[5] Ganesan S, Volodina O, Pearce SC, Gabler NK, Baumgard LH, Rhoads RP, Selsby JT (2017). Acute heat stress activated inflammatory signaling in porcine oxidative skeletal muscle. Physiol Rep.

[6] Hao Y, Cui Y, Gu X (2016). Genome-wide DNA methylation profiles changes associated with constant heat stress in pigs as measured by bisulfite sequencing. Sci Rep.

[7] Kamanga-Sollo E, Pampusch M, White M, Hathaway M, Dayton W (2011). Effects of heat stress on proliferation, protein turnover, and abundance of heat shock protein messenger ribonucleic acid in cultured porcine muscle satellite cells. J Anim Sci.

[8] Johnson JS, Stewart KR, Safranski TJ, Ross JW, Baumgard LH (2020). In utero heat stress alters postnatal phenotypes in swine. Theriogenology.

[9] Johnson JS, Fernandez MVS, Patience JF, Ross JW, Gabler NK, Lucy MC (2015). Effects of in utero heat stress on postnatal body composition in pigs: II. Finishing phase. J Anim Sci.

[10] Boddicker RL, Seibert JT, Johnson JS, Pearce SC, Selsby JT, Gabler NK, Lucy MC, Safranski TJ, Rhoads RP, Baumgard LH, Ross JW (2014). Gestational heat stress alters postnatal offspring body composition indices and metabolic parameters in pigs. PLoS One.

[11] Tuell JR, Nondorf MJ, Maskal JM, Johnson JS, Kim YHB (2021). Impacts of in utero heat stress on carcass and meat quality traits of market weight gilts. Animals..

[12] Liu F, Ford EM, Morrison RS, Brewster CJ, Henman DJ, Smits RJ, Zhao W, Cottrell JJ, Leury BJ, Dunshea FR, Bell AW (2020). The greater proportion of born-light progeny from sows mated in summer contributes to increased carcass fatness observed in spring. Animals..

[13] Bell AW (2006). Prenatal programming of postnatal productivity and health of livestock: a brief review. Aust J Exp Agric.

[14] Bell AW, Wilkening RB, Meschia G (1987). Some aspects of placental function in chronically heat-stressed ewes. J Dev Physiol.

[15] Zhao W, Liu F, Bell AW, Le HH, Cottrell JJ, Leury BJ, et al. Controlled elevated temperatures during early-mid gestation cause placental insufficiency and implications for fetal growth in pregnant pigs. Sci Rep. 2020;10:20677.10.1038/s41598-020-77647-1. 10.1038/s41598-020-77647-1 PMC7691357 33244103

[16] Wigmore PM, Stickland NC (1983). Muscle development in large and small pig fetuses. J Anat.

[17] Zhao W, Liu F, Marth CD, Green MP, Le HH, Leury BJ (2021). Maternal heat stress alters expression of genes associated with nutrient transport activity and metabolism in female placentae from mid-gestating pigs. Int J Mol Sci.

[18] Andrews S (2010). FastQC: a quality control tool for high throughput sequence data.

[19] Kim D, Langmead B, Salzberg SL (2015). HISAT: a fast spliced aligner with low memory requirements. Nat Methods.

[20] Anders S, Pyl PT, Huber W (2015). HTSeq—a Python framework to work with high-throughput sequencing data. Bioinformatics..

[21] Robinson MD, McCarthy DJ, Smyth GK (2010). edgeR: a Bioconductor package for differential expression analysis of digital gene expression data. Bioinformatics..

[22] R Core Team. R: a language and environment for statistical computing. Vienna: R Foundation for Statistical Computing; 2013. https://www.R-project.org/. Accessed 08 Apr 2022.

[23] Robinson MD, Oshlack A. A scaling normalization method for differential expression analysis of RNA-seq data. Genome Biol. 2010;11:R25. 10.1038/s41598-020-77647-1. 10.1186/gb-2010-11-3-r25 PMC2864565 20196867

[24] Raudvere U, Kolberg L, Kuzmin I, Arak T, Adler P, Peterson H, Vilo J (2019). g: Profiler: a web server for functional enrichment analysis and conversions of gene lists (2019 update). Nucleic Acids Res.

[25] Subramanian A, Tamayo P, Mootha VK, Mukherjee S, Ebert BL, Gillette MA, Paulovich A, Pomeroy SL, Golub TR, Lander ES, Mesirov JP (2005). Gene set enrichment analysis: a knowledge-based approach for interpreting genome-wide expression profiles. Proc Natl Acad Sci U S A.

[26] Sergushichev A (2016). An algorithm for fast preranked gene set enrichment analysis using cumulative statistic calculation. Bio Rxiv.

[27] Horak ER, Klenk N, Leek R, Lejeune S, Smith K, Stuart N (1992). Angiogenesis, assessed by platelet/endothelial cell adhesion molecule antibodies, as indicator of node metastases and survival in breast cancer. Lancet..

[28] Stenhouse C, Hogg CO, Ashworth CJ (2018). Associations between fetal size, sex and placental angiogenesis in the pig. Biol Reprod.

[29] Gionbelli T, Veloso C, Rotta P, Valadares Filho SC, Carvalho B, Marcondes M (2018). Foetal development of skeletal muscle in bovines as a function of maternal nutrition, foetal sex and gestational age. J Anim Physiol an N.

[30] Rudolph TE, Mayorga EJ, Roths M, Rhoads RP, Baumgard LH, Selsby JT (2021). The effect of Mitoquinol (MitoQ) on heat stressed skeletal muscle from pigs, and a potential confounding effect of biological sex. J Therm Biol.

[31] Brown LD, Hay WW (2016). Impact of placental insufficiency on fetal skeletal muscle growth. Mol Cell Endocrinol.

[32] Sartorelli V, Caretti G (2005). Mechanisms underlying the transcriptional regulation of skeletal myogenesis. Curr Opin Genet Dev.

[33] Bailey P, Downes M, Lau P, Harris J, Chen SL, Hamamori Y, Sartorelli V, Muscat GEO (1999). The nuclear receptor corepressor N-CoR regulates differentiation: N-CoR directly interacts with MyoD. Mol Endocrinol.

[34] MacLellan WR, Xiao G, Abdellatif M, Schneider MD (2000). A novel Rb-and p300-binding protein inhibits transactivation by MyoD. Mol Cell Biol.

[35] Lee GE, Kim JH, Taylor M, Muller MT (2010). DNA methyltransferase 1-associated protein (DMAP1) is a co-repressor that stimulates DNA methylation globally and locally at sites of double strand break repair. J Biol Chem.

[36] Greenwood PL, Slepetis RM, Bell AW, Hermanson JW (1999). Intrauterine growth retardation is associated with reduced cell cycle activity, but not myofibre number, in ovine fetal muscle. Reprod Fertil Dev.

[37] Albrecht ED, Pepe GJ (2010). Estrogen regulation of placental angiogenesis and fetal ovarian development during primate pregnancy. Int J Dev Biol.

[38] Franck-Lissbrant I, Häggström S, Damber J-E, Bergh A (1998). Testosterone stimulates angiogenesis and vascular regrowth in the ventral prostate in castrated adult rats. Endocrinology..

[39] Colenbrander B, De Jong F, Wensing C (1978). Changes in serum testosterone concentrations in the male pig during development. Reproduction..

[40] Lin J, Tarr PT, Yang R, Rhee J, Puigserver P, Newgard CB, Spiegelman BM (2003). PGC-1β in the regulation of hepatic glucose and energy metabolism. J Biol Chem.

[41] Schoonjans K, Staels B, Auwerx J (1996). The peroxisome proliferator activated receptors (PPARS) and their effects on lipid metabolism and adipocyte differentiation. Biochim Biophys Acta Lipids Lipid Metab.

[42] Lin J, Puigserver P, Donovan J, Tarr P, Spiegelman BM (2002). Peroxisome proliferator-activated receptor γ coactivator 1β (PGC-1β), a novel PGC-1-related transcription coactivator associated with host cell factor. J Biol Chem.

[43] Lin J, Yang R, Tarr PT, Wu P-H, Handschin C, Li S, Yang W, Pei L, Uldry M, Tontonoz P, Newgard CB, Spiegelman BM (2005). Hyperlipidemic effects of dietary saturated fats mediated through PGC-1β coactivation of SREBP. Cell..

[44] Chinetti G, Fruchart J-C, Staels B (2000). Peroxisome proliferator-activated receptors (PPARs): nuclear receptors at the crossroads between lipid metabolism and inflammation. Inflamm Res.

[45] Kim JB, Spiegelman BM (1996). ADD1/SREBP1 promotes adipocyte differentiation and gene expression linked to fatty acid metabolism. Genes Dev.

[46] Heng J, Tian M, Zhang W, Chen F, Guan W, Zhang S (2019). Maternal heat stress regulates the early fat deposition partly through modification of m 6 a RNA methylation in neonatal piglets. Cell Stress Chaperones.

[47] Karunaratne J, Ashton C, Stickland N (2005). Fetal programming of fat and collagen in porcine skeletal muscles. J Anat.

[48] Sanes JR (2003). The basement membrane/basal lamina of skeletal muscle. J Biol Chem.

[49] Péterfi Z, Donkó A, Orient A, Sum A, Prókai A, Molnár B, Veréb Z, Rajnavölgyi É, Kovács KJ, Müller V, Szabó AJ, Geiszt M (2009). Peroxidasin is secreted and incorporated into the extracellular matrix of myofibroblasts and fibrotic kidney. Am J Clin Pathol.

[50] Sordella R, Jiang W, Chen G-C, Curto M, Settleman J (2003). Modulation of rho GTPase signaling regulates a switch between adipogenesis and myogenesis. Cell..

[51] Artaza JN, Bhasin S, Magee TR, Reisz-Porszasz S, Shen R, Groome NP, Fareez MM, Gonzalez-Cadavid NF (2005). Myostatin inhibits myogenesis and promotes adipogenesis in C3H 10T (1/2) mesenchymal multipotent cells. Endocrinology..

[52] Du M, Yan X, Tong JF, Zhao J, Zhu MJ (2010). Maternal obesity, inflammation, and fetal skeletal muscle development. Biol Reprod.

[53] Bradley JR, Pober JS (2001). Tumor necrosis factor receptor-associated factors (TRAFs). Oncogene..

[54] Burns K, Clatworthy J, Martin L, Martinon F, Plumpton C, Maschera B, Lewis A, Ray K, Tschopp J, Volpe F (2000). Tollip, a new component of the IL-1RI pathway, links IRAK to the IL-1 receptor. Nat Cell Biol.

[55] Gottipati S, Rao NL, Fung-Leung WP (2008). IRAK1: a critical signaling mediator of innate immunity. Cell Signal.

[56] Johnson JS, Maskal JM, Duttlinger AW, Kpodo KR, McConn BR, Byrd CJ, et al. In utero heat stress alters the postnatal innate immune response of pigs. J Anim Sci. 2020;98(12):skaa356. 10.1093/jas/skaa356. 10.1093/jas/skaa356 PMC7739886 33159520

